# Supplementary material for: Luck of the draw: Role of chance in the assignment of medicare readmissions penalties
Source: PLoS One. 2021 Dec 21;16(12):e0261363. doi: 10.1371/journal.pone.0261363 (PMC8691630; doi:10.1371/journal.pone.0261363)
Supplement: S1 File — (DOCX) [file pone.0261363.s001.docx]

**Supporting Information**

**Table of Contents**

Condition Specific Co-Morbidities (page 2)

Table S1. Co-Morbidity Risk Adjusters for AMI (page 2)

Table S2. Co-Morbidity Risk Adjusters for CHF (page 3)

Table S3. Co-Morbidity Risk Adjusters for Pneumonia (page 4)

Table S4. Co-Morbidity Risk Adjusters for COPD (page 5)

Table S5. Co-Morbidity Risk Adjusters for TKA/THA (page 6)

**Condition Specific Co-Morbidities**

The HRRP risk-adjusts for patient co-morbidities when estimating the Excess Readmission Ratios for each of the 5 conditions evaluated in FY2015. The risk adjustment variables are described fully in the appendices of the **2015 Condition-Specific Measures Updates and Specifications Report Hospital-Level 30-Day Risk-Standardized Readmission Measures** report prepared for Medicare by the Yale New Haven Health Services Corporation/Center for Outcomes Research & Evaluation (YNHHSC/CORE) (published in March 2015). The risk-adjusters are largely described by their condition categories (CCs), which are groupings of ICD-9-CM diagnosis codes in clinically relevant categories, from the Hierarchical Condition Categories (HCCs) system.

We created co-morbidity dichotomous indicators (yes/no) using the first 10 diagnoses on each index admission claim, employing the same CCs outlined by Medicare. Some CCs are considered complications when appearing on the index admission, and thus were excluded from our list. We used the first 10 diagnoses only to avoid complications from the switch from 10 to 25 diagnoses recorded on a claim.^[[1]](#footnote-1)^The following describes the codes and co-morbidities we included for each FY2015 HRRP condition.

**Table S1. Co-Morbidity Risk Adjusters for AMI**

| **Codes Used** | **Co-Morbidity Indicator** |
| --- | --- |
| ICD-9 codes V45.82, 00.66, 36.06, 36.07 | History of Percutaneous Transluminal Coronary Angioplasty (PTCA) |
| ICD-9 codes V45.81, 36.10–36.16 | History of Coronary Artery Bypass Graft (CABG) |
| ICD-9 codes 410.00-410.12 | Anterior myocardial infarction |
| ICD-9 codes 410.20-410.62 | Other location of myocardial infarction |
| CC 1, 3-5 | History of infection |
| CC 7 | Metastatic cancer or acute leukemia |
| CC 8-12 | Cancer |
| CC 15, 16, 18-20, 119-120 | Diabetes mellitus (DM) or DM complications |
| CC 21 | Protein-calorie malnutrition |
| CC 22 | Disorders of fluid/electrolyte/acid-base |
| CC 47 | Iron deficiency or other specified anemias and blood disease |
| CC 49-50 | Dementia and other specified brain disorders |
| CC 67-69 | Hemiplegia, paraplegia, paralysis, functional disability |
| CC 83 | Angina pectoris/old myocardial infarction |
| CC 84 | Coronary atherosclerosis |
| CC 86 | Valvular or rheumatic heart disease |
| CC 97-99, 103 | Cerebrovascular disease |
| CC 108 | Chronic obstructive pulmonary disease (COPD) |
| CC 110 | Asthma |
| CC 113 | Pneumonia |
| CC 136 | Other urinary tract disorders |
| CC 149 | Decubitus ulcer or chronic skin ulcer |

**Table S2. Co-Morbidity Risk Adjusters for CHF**

| **Codes Used** | **Co-Morbidity Indicator** |
| --- | --- |
| ICD-9 codes V45.81, 36.10–36.16 | History of Coronary Artery Bypass Graft (CABG) |
| CC 7 | Metastatic cancer or acute leukemia |
| CC 8-12 | Cancer |
| CC 15, 16, 18-20, 119-120 | Diabetes mellitus (DM) or DM complications |
| CC 21 | Protein-calorie malnutrition |
| CC 22 | Disorders of fluid/electrolyte/acid-base |
| CC 25-27, 29-30 | Liver or biliary disease |
| CC 36 | Other gastrointestinal disorders |
| CC 44 | Severe hematological disorders |
| CC 47 | Iron deficiency or other unspecified anemias and blood disease |
| CC 49-50 | Dementia or other specified brain disorders |
| CC 51-53 | Drug/alcohol abuse/dependence/psychosis |
| CC 54-56 | Major psychiatric disorders |
| CC 58 | Depression |
| CC 60 | Other psychiatric disorders |
| CC 67-69 | Hemiplegia, paraplegia, paralysis, functional disability |
| CC 83-84 | Coronary atherosclerosis or angina |
| CC 86 | Valvular or rheumatic heart disease |
| CC 94 | Other or unspecified heart disease |
| CC 108 | Chronic obstructive pulmonary disease (COPD) |
| CC 109 | Fibrosis of lung or other chronic lung disorders |
| CC 110 | Asthma |
| CC 113 | Pneumonia |
| CC 136 | Other urinary tract disorders |
| CC 149 | Decubitus ulcer or chronic skin ulcer |

**Table S3. Co-Morbidity Risk Adjusters for Pneumonia**

| **Codes Used** | **Co-Morbidity Indicator** |
| --- | --- |
| ICD-9 codes V45.81, 36.10–36.16 | History of Coronary Artery Bypass Graft (CABG) |
| CC 1, 3-5 | History of infection |
| CC 7 | Metastatic cancer or acute leukemia |
| CC 8 | Lung, upper digestive tract, and other severe cancers |
| CC 9-10 | Other major cancers |
| CC 15, 16, 18-20, 119-120 | Diabetes mellitus (DM) or DM complications |
| CC 21 | Protein-calorie malnutrition |
| CC 22 | Disorders of fluid/electrolyte/acid-base |
| CC 36 | Other gastrointestinal disorders |
| CC 44 | Severe hematological disorders |
| CC 47 | Iron deficiency or other unspecified anemias and blood disease |
| CC 49-50 | Dementia or other specified brain disorders |
| CC 51-53 | Drug/alcohol abuse/dependence/psychosis |
| CC 54-56 | Major psychiatric disorders |
| CC 60 | Other psychiatric disorders |
| CC 67-69 | Hemiplegia, paraplegia, paralysis, functional disability |
| CC 83-84 | Coronary atherosclerosis or angina |
| CC 86 | Valvular or rheumatic heart disease |
| CC 108 | Chronic obstructive pulmonary disease (COPD) |
| CC 109 | Fibrosis of lung or other chronic lung disorders |
| CC 110 | Asthma |
| CC 113 | Pneumonia |
| CC 115 | Other lung disorders |
| CC 136 | Other urinary tract disorders |
| CC 149 | Decubitus ulcer or chronic skin ulcer |
| CC 157 | Vertebral fractures |
| CC 162 | Other injuries |

**Table S4. Co-Morbidity Risk Adjusters for COPD**

| **Codes Used** | **Co-Morbidity Indicator** |
| --- | --- |
| ICD-9 codes 93.90, 96.70, 96.71, 96.72 | History of mechanical ventilation |
| ICD-9 codes 327.20, 327.21, 327.23, 327.27, 327.29, 780.51, 780.53, 780.57 | Sleep apnea |
| CC 1, 3-5 | History of infection |
| CC 7 | Metastatic cancer or acute leukemia |
| CC 8 | Lung, upper digestive tract, and other severe cancers |
| CC 9-11 | Lymphatic, head and neck, brain, and other major cancers; breast, colorectal and other cancers and tumors; other respiratory and heart neoplasms |
| CC 12 | Other digestive and urinary neoplasms |
| CC 15, 16, 18-20, 119-120 | Diabetes mellitus (DM) or DM complications |
| CC 21 | Protein-calorie malnutrition |
| CC 22 | Disorders of fluid/electrolyte/acid-base |
| CC 24 | Other endocrine/metabolic/nutritional disorders |
| CC 32 | Pancreatic disease |
| CC 36 | Other gastrointestinal disorders |
| CC 44 | Severe hematological disorders |
| CC 47 | Iron deficiency or other unspecified anemias and blood disease |
| CC 49-50 | Dementia or other specified brain disorders |
| CC 51-52 | Drug/alcohol psychosis or dependence |
| CC 54-56 | Major psychiatric disorders |
| CC 58 | Depression |
| CC 59 | Anxiety disorders |
| CC 60 | Other psychiatric disorders |
| CC 67-69 | Hemiplegia, paraplegia, paralysis, functional disability |
| CC 71 | Polyneuropathy |
| CC 83-84 | Chronic atherosclerosis or angina |
| CC 89 | Hypertensive heart and renal disease or encephalopathy |
| CC 94 | Other or unspecified heart disease |
| CC 109 | Fibrosis of lung or other chronic lung disorders |
| CC 113 | Pneumonia |
| CC 149 | Decubitus ulcer or chronic skin ulcer |
| CC 157 | Vertebral fractures |

**Table S5. Co-Morbidity Risk Adjusters for TKA/THA**

| **Codes Used** | **Co-Morbidity Indicator** |
| --- | --- |
| Any procedure | Two or more procedures during admission |
| ICD-9 code 755.63 | Other congenital deformity of hip (joint) |
| ICD-9 codes 716.15, 716.16 | Post traumatic osteoarthritis |
| ICD-9 code 278.01 | Morbid obesity |
| CC 1, 3-5 | History of infection |
| CC 7 | Metastatic cancer or acute leukemia |
| CC 8-12 | Cancer |
| CC 15, 16, 18-20, 119-120 | Diabetes mellitus (DM) or DM complications |
| CC 21 | Protein-calorie malnutrition |
| CC 22 | Disorders of fluid/electrolyte/acid-base |
| CC 38 | Rheumatoid arthritis and inflammatory connective tissue disease |
| CC 44 | Severe hematological disorders |
| CC 49, 50 | Dementia or other specified brain disorders |
| CC 54-56 | Major psychiatric disorders |
| CC 67-69 | Hemiplegia, paraplegia, paralysis, functional disability |
| CC 71 | Polyneuropathy |
| CC 83-84 | Coronary atherosclerosis or angina |
| CC 89, 91 | Hypertension |
| CC 108 | Chronic obstructive pulmonary disease (COPD) |
| CC 149 | Decubitus ulcer or chronic skin ulcer |
| CC 166 | Major symptoms, abnormalities |

1. Ody C, Msall L, Dafny LS, Grabowski DC, Cutler DM. Decreases in readmissions credited to Medicare’s program to reduce hospital readmissions have been overstated. Health Affairs. 2019 Jan 1;38(1):36-43. [↑](#footnote-ref-1)
