# Supplementary material for: Luck of the draw: Role of chance in the assignment of medicare readmissions penalties
Source: PLoS One. 2021 Dec 21;16(12):e0261363. doi: 10.1371/journal.pone.0261363 (PMC8691630; doi:10.1371/journal.pone.0261363)
Supplement: Attachment — Submitted filename: Responses.docx [file pone.0261363.s002.docx]

**Journal Requirments:**

**1. Please ensure that your manuscript meets PLOS ONE's style requirements, including those for file naming.**

We confirm that our revision meets PLOS ONE’s style requirements. Notably, we no longer include our exhibits at the end of the manuscript, but include them in an appropriate location after their first mention, embedded in the text. If we have missed a style requirement that needs to be resolved before evaluating out revision, then please let us know and we will fix it ASAP.

**2. Please include your actual numerical p-values in Table 4**

In our revised manuscript we have included p-values in Table 4. Also, in responding to reviewer 2’s comments about the assumptions of this model we realized we hadn’t employed robust standard errors in this model. In our revision, we changed this to include robust standard errors.

While making this change we found that some of the estimates in our exhibits were from earlier versions of our manuscript. We thoroughly reviewed our programs and output, and have updated all tables with our final results. Substantively, there’s little difference with the estimates from our initial submission: Tables 1 and 2 are exactly the same, Figure 1’s and Table 3’s values were slightly different (almost no difference), and Table 4’s estimates were smaller but consistent in size and significance with our prior findings. The one exception was the share of admissions from Medicare, which is in the same direction but smaller and no longer significant. We have adjusted our language in the text to reflect these latest estimates and we apologize for not having caught this in our original submission.

Also, we wanted to highlight here that we added more details to our simulated penalty Methods section, which we noticed were absent from our initial submission. The final part of this section now reads:

Finally, we used the simulated readmissions to calculate the simulated ERR for each hospital and condition, assigned a simulated penalty status, and calculated a simulated total penalty size for FY2015, following the same methods as for the recreation of the FY2015 penalties. We replicated the above steps 1,000 times for each hospital, representing 1,000 hypothetical fiscal years during which penalties could be assigned. *To correct for additive bias (i.e., “bootstrap bias”) in our simulated penalties introduced from repeatedly sampling from the observed data, we added a constant to each simulated penalty equal to the difference between the mean of the hospital’s simulated penalties and their actual penalty assignment This had the effect of centering each hospital’s distribution of simulated penalties on the actual penalty assigned by HRRP.*

**3. In your ethics statement in the Methods section and in the online submission form, please provide additional information about the data used in your retrospective study. Specifically, please ensure that you have discussed whether all data were fully anonymized before you accessed them and/or whether the IRB or ethics committee waived the requirement for informed consent. If patients provided informed written consent to have data from their medical records used in research, please include this information.**

We have clarified our ethics in our Methods section by adding the following at the beginning of the section on Analysis:

*University of Minnesota IRB granted approval for this study. Patient informed consent was not required given the data was de-identified.*

**4. Thank you for providing the date(s) when patient medical information was initially recorded. Please also include the date(s) on which your research team accessed the databases/records to obtain the retrospective data used in your study.**

We have clarified this in our revised manscript by adding the following to the beginning of the Methods-Analysis section:

*We conducted all analyses using SAS version 9.4 and Stata version 14 over the period 2018-19.*

**Additional Editor Comments:**

**1. Please follow PLOS ONE formatting requirements on exhibits. For example, all tables must be embedded within the text after the paragraph where they are first mentioned. Figure captions must also be embedded within the text.**

Thank you for pointing this out. We have embedded the exhibits in the text after their first mention, per PLOS ONE formatting requirements.

**2. In Introduction, in 1-2 sentences, please describe the Medicare program. Please bear in mind that PLOS ONE has an international readership and they may not be highly familiar with US healthcare programs. Similarly, add more detail on HRRP, eg, possible penalties, 30-day “all-cause” readmissions, evaluation period, etc.**

We have changed the first paragraph in the introduction to include a sentence on what Medicare is, it now reads:

Pay-for-performance programs are one strategy used by health plans to improve the efficiency and quality of care delivered to beneficiaries. Under such programs, providers are often compared against their peers in order to win bonuses or face penalties in payment. Yet luck has the potential to impact payers’ ability to make accurate and fair assessments of performance. *Medicare, the national health insurance program in the United States for Americans aged 65 years and older, has developed and employed a suite of pay-for-performance initiatives since the passage of the Affordable Care Act in 2010.*

**3. Pg. 5: What is the size of the study population? State how many hospitals were included and the number and % missing.**

In the revised methods we have clarified the size of the study population, which are the 3,130 acute care hospitals included in the FY2015 HRRP program that cared for the 4,159,463 discharges with one of the 5 conditions under risk as part of the HRRP program. In addition, we say that 3.8% of these hospitals were missing AHA characteristics.

**4. Pg. 6: Write out “CMS” at 1st mention. Either write out the condition-specific comorbidities or list them in Supporting Information. Clarify how minor vs. major teaching hospitals were defined. “number of beds” instead of “bed size”. Briefly describe DSH. Add “and” in front of “Medicare share” and briefly define it. Finally, clarify why these particular hospital characteristics were selected.**

Thank you for asking us to unpack the CMS abbreviation. We realized that we only used it a few times, and for the most part say “Medicare” where we could have said “CMS”. Instead of spelling out CMS at first mention, we replace all instances of CMS with Medicare. We feel this simplifies things for readers.

Regarding the additional details on the hospital characteristics we selected for our analysis, we edited this paragraph in the revised draft. It now reads:

We selected hospital characteristics that describe less-modifiable “structural” features to better understand if luck affects some hospitals more than others. These included ownership (not-for-profit, for-profit, or government), teaching status (major teaching, which are hospitals that are members of the Association of American Medical Colleges Council of Teaching Hospitals, minor teaching, which are hospitals with other teaching affiliations/accreditations, and non-teaching hospitals), number of beds (<100, 100-199, 200-399, and ≥400), location (non-metropolitan, metropolitan), census region (Northeast, South, Midwest, West), safety-net status (based on the hospital’s Disproportionate Share Hospital (DSH) payment percentage, which refers to the relative share of means tested care they delivered compared to their total; yes, which includes hospitals with 80% or more, no, which are hospitals with less than 80%), and Medicare’s share of annual hospital admissions (<40%, ≥40%).

**5. Pg. 7: Write out “ERR” at 1st mention and define what it is.**

Thanks for pointing this out, we have written it out at first mention in our revised draft.

**6. Pg 8: Add more detail on the regression variables, eg, length of stay is number of inpatient nights, what were the age groups and seasons? Clarify why the variables were selected for the regression modeling. Patient race is mentioned but not listed in the 1st paragraph nor defined. Was ethnicity included? To clarify, were any other patient characteristics, eg, income or other socio-economic status variables, included? Finally, as reviewers suggest, please provide more detail on the modeling and report the regression estimates in Supporting Information.**

Due to the number of iterations (1000 bootstrapped simulation models, over 5 different conditions), including ERR regression results as an exhibit in our manuscript is not realistic. However, we have added more details to the methods on the variables we included in our regressions, as well as more details on what type of regressions we ran. These sections now read:

First, we used regression models to predict the risk of readmission for each eligible index discharge as a function of condition, length of stay (i.e., number of inpatient nights), age group (65-69, 70-74, 75-79, 80-84, 85-89, 90+), sex, dual-eligibility for Medicaid, race/ethnicity (white, black, Asian, Hispanic, other race), season of discharge month (winter, spring, summer, fall), condition-specific co-morbidities (see Supporting Information Tables S1-S5), and hospital-level fixed effects. In these analyses, we modeled the risk of readmission not only as a function of the HRRP risk adjusters, but also as a function of other observable patient characteristics such as dual eligibility and race.

**7. Pg. 8: “between 0 and 1”.**

We made this change.

**8. Pg. 9: “Then, we graphed…”. “we calculated the average penalty…”. “In addition, we calculated…”. Be consistent in using commas for “i.e.,”.**

We added the missing comma, thanks for catching this.

**9. Pg. 10: add commas around “by design”. Also, here and elsewhere in the text, be consistent in using either “within-hospital” or “within hospital”. Add a comma between “penalties” and “we”.**

We added the requested commas on this page, and we checked the entire manuscript for instances of “within-hospital” or “within hospital”, switching every instance to the former. We did the same with “across-hospital” when it was similarly referring to variation.

**10. Pg. 11: add a comma between “penalties” and “we”. Please specify these SES measures on pg. 8, 1st paragraph.**

We made this change, as well as a similar one on page 13. Regarding SES, we clarified on page 11 that this was referring to dual-eligibility for Medicaid.

**11. Pg. 12: add “and” between “FY2015” and “it”. Extra space in “28.8 %”.**

We made these changes, thank you.

**12. Pg. 13: use “1,000” to be consistent.**

We made this change.

**13. Pg. 14: “In our analysis…”. “because penalties” instead of “since”. Here and elsewhere, avoid contractions like “it’s”, “don’t”, etc. Use a comma instead of semi-colon in front of “generating”.**

We made these changes. We only found 2 contractions in our text, which we uncontracted.

**14. Pg. 15: avoid starting sentences with “And”. “variation in the patient population…”.**

We made these edits, changing the sentence starting with “And” to

Second, this is an inefficient policy. Penalties signal hospitals on whether to change their practices to lower readmission. If they are based on chance, then low performers may not know when to change and high performers may change when they do not need to. This means that hospital practice changes stemming from the HRRP may not always be an improvement, as some evidence indicates mortality has been higher for heart failure patients since the start of the program.[11] Policy makers need to consider the tradeoff the HRRP appears to be making: are the savings generated from randomly distributing penalties worth the risk of incentivizing changes in care delivery in ways that could adversely impact patient access and welfare.

**15. Pg. 16: add “the” in front of “Hospital-Acquired…”.**

We made this change, thank you.

**16. Figure 1: suggest adding the % symbol to the x-axis labels. Also, use consistent terminology in figure title vs. text, ie, “cumulative distribution” vs. “cumulative density function”. The y-axis title in the caption should be embedded in the figure (and the caption must be embedded within the text). Please confirm that PLOS ONE requirements regarding figures have been followed (https://journals.plos.org/plosone/s/figures).**

We change the figure title to “Cumulative Distribution” and have embedded the y-axis in the figure, which is now located in the text when it is first mentioned, per PLOS ONE guidelines.

**17. Conclusions: add a comma between “commonplace” and “care”.**

We have made this change.

**18. Please include an Acknowledgements section prior to References.**

We have added an acknowledgements section in our revised draft.

**19. References must follow PLOS ONE requirements, eg, citations in text should use square brackets, journal articles must use NCBI abbreviations, #8 has incorrect formatting of author names, etc.**

We have edited the references per PLOS ONE requirements.

**20. If you include Supporting Information, please follow PLOS ONE requirements.**

We have added Supporting Information as part of our revision that follows PLOS ONE requirements.

**Reviewer #1 Comments:**

1. **This study used 2010-2013 Medicare claims data and a simulation model to examine the role of luck on 30-day unplanned readmission among the Medicare beneficiaries whose primary diagnoses during index admission (IA) were under the Hospital Readmission Reduction Program. The authors found that luck played a significant role in hospitals’ readmission performance.**

Thank you for the brief summary of our manuscript.

2. **The authors may need clearer logic to justify their assumptions regarding the two mechanisms through which luck would affect hospital readmission. Although the authors emphasized the role of luck (i.e., random variations), their study design did not clearly differentiate (i) real randomness, as demonstrated in their mechanisms, from (ii) the determinants of IA (e.g., various health services utilization prior to 30 days of IA) and readmission (e.g., number of procedures during the IA, discharge status of IA) that were identified in relevant literature and available in the Medicare claims data but were not adjusted for their study. For example, more than two thirds of readmissions occurred for the different primary diagnoses from the IA, but a substantial proportion of patients used various post-discharge care related with these diagnoses, which significantly decreased readmission (e.g., Dharmarajan et al., JAMA, 2013; Tak et al., JGIM, 2019).**

We appreciate the reviewer’s comment, and we thank them for pointing out these important papers showing unplanned readmissions occur more often for diagnoses different from their indexed admission diagnosis. Our goal wasn’t to suggest new adjusters for the HRRP. Our goal was to simulate the penalty generating process Medicare conducts, where adjustment includes a limited set of demographics and comorbidities. And while it’s likely these additional factors play a role in unplanned readmission, Medicare does not adjust for them when evaluating hospital readmissions leaving open a bigger role for luck to play in setting penalties. This was the basic point we were trying to make.

We have added more language in our discussion to say that luck plays a larger role in readmission penalties because Medicare doesn’t adjust for key factors such as post-acute care use or contrasting diagnoses, and we have cited the articles you’ve suggested. This section now reads:

Prior research has documented that variation in readmission rates is driven by differences in patient case-mix.[3] Important determinants of readmission such as socioeconomic status [12-14] and community disadvantage [15,16] have not, until recently, been accounted for in HRRP penalty assignment—a shortcoming that has left hospitals serving these communities accountable for their locations’ above average readmission risk.[17-19] In response to these criticisms, Medicare began (starting in FY2019) comparing hospitals with similar proportions of disadvantaged patients when calculating HRRP penalties. While these changes could change hospitals’ expectations of being penalized, our analysis suggests that these changes will not diminish the role of luck in determining readmission penalties as we find that variation in the patient population does not play an important role in explaining variation in excess readmission risk. *Rather, our analysis supports other research that has found post-discharge care patterns play an important role in determining unplanned readmissions, and by not including these factors in their risk-adjustment Medicare leaves open a bigger role for luck in assigning penalties.[20,21]*

3. **In Analysis (pp.7-8), the authors simply stated that they used “regression models” to estimate the predicted number of readmissions for each hospital and risk of readmission for each eligible index discharge. Please specify what regression models the authors used.**

We have revised the Methods-Analysis section to clarify what kind of regression models we used. This section now reads:

First, we identified eligible index admissions and readmissions from July 2010 through June 2013 for each hospital and condition. We estimated the predicted readmission rate for each hospital and condition by taking the mean of the probability of readmission for each of their discharges with a given condition. Readmission probabilities were estimated with a linear probability regression model of unplanned 30-day readmission on HRRP risk-adjustment variables (age, sex, and comorbidities) and hospital-level random effects. We employed linear probability models to minimize the computational burden and time involved with running non-linear models.

Next, we estimated the expected readmission rate for each hospital and condition using another set of linear probability regression models that regressed unplanned 30-day readmission on the HRRP risk-adjustment variables but not hospital-level random effects. For each hospital and condition, we calculated the ERR by dividing the predicted readmissions by the expected readmissions.

4. **Briefly explain the criteria for the eligible IA (e.g., a discharge status of not being transferred to another acute care facility) and for the 30-day all-cause unplanned readmission (e.g., first readmission only when there were multiple readmissions within 30 days), and cite references.**

To provide more clarity for readers, we have edited our methods section to include more details on discharge eligibility and readmission. These sections now read:

Study hospitals included 3,130 acute care hospitals that were subject to the HRRP in FY2015. The underlying study population included 4,159,463 discharges from adults age 65 or older who had an index discharge between July 2010 and June 2013 for one of five conditions targeted by the HRRP: acute myocardial infarction (AMI), heart failure (HF), pneumonia, chronic obstructive pulmonary disease (COPD), and elective total hip and knee arthroplasty (THA and TKA). To qualify as an index discharge, the beneficiary had to be discharged alive into the community (i.e., not transferred to a post-acute care facility) and had to be continuously enrolled in Medicare Parts A and B (i.e., coverage for inpatient and outpatient/professional services respectively) from 12 months prior to the end of their admission, and continuously enrolled in Part A through the post-discharge 30 day evaluation window for readmission.[5,6] Patients that transferred between hospitals during their indexed admission are attributed to the final hospital that discharged the patient. Medicare used discharges from this three-year period to assign penalties in FY2015.

5. **Please write the p-values for the correlation coefficients (and other statistical analyses, if applicable).**

The p-value for the 0.81 correlation coefficient was < .001. We have added this in the methods text.

6. **Please spell out what ERR stands for.**

We have made this change, thank you.

**Reviewer #2 Comments:**

1. **The authors present interesting results from a simulation study of Medicare readmission penalties. Their simulations were designed to assess two sources of randomness in readmission: 1) who gets admitted to the hospital in the first place; and 2) post-discharge occurrences (for who gets readmitted). Authors call this randomness "luck" and assess how luck impacts the penalties that get imposed. They find that most of the variability in penalties can be attributed to the randomness in events after discharge. They also found that larger hospitals, teaching hospitals, safety-net hospitals and hospitals with a larger share of Medicare patients have less variability in penalties (so a lower contribution of luck). The manuscript will be strengthened if the authors consider the following points.**

We thank the reviewer for the concise summary of our paper, and for their helpful comments below. We believe the manuscript has been strengthened because of them.

2. **Authors use ANOVA to assess the between versus within hospital variability. Based on the author's description, it is not entirely clear what is being done here. Is the penalty the outcome with factors of hospital and maybe hypothetical fiscal year? Are the assumptions of ANOVA met?**

We have revised this section of the methods to better explain our approach as a decomposition of variance only (Stata’s xtsum command). We no longer use ANOVA, which as the reviewer points out has assumptions. This section now reads:

In contrast to within-hospital variation in simulated penalties, between-hospital variation in penalties reflects variation in penalties due to luck as well as other factors such as quality of care. Therefore, as another measure to quantify the role of luck in hospital readmission penalties, we decomposed the variance by within-hospital variation in penalties (which represents luck only) and between-hospital variation in penalties (which represents luck and other factors). We assessed the size of the within-hospital variation relative to the between-hospital variation as a proportion.

3. **Authors use multivariate regression to assess how different factors might be associated with "luck", quantified by the standard deviation in the penalties across the 1000 hypothetical fiscal years for each hospital. Were the assumptions of the linear regression model met for these data? Were there any issues of collinearity (or substantial overlap) among the factors considered in the model?**

We thank the reviewer for asking about our model assumptions. We have clarified in our revised methods why we selected the factors we included in our model, *“We selected hospital characteristics that describe less-modifiable “structural” features to better understand if luck affects some hospitals more than others.”* Because these factors are structural, and therefore exogenous, it is unlikely they are correlated with our error term. This was our basic assumption. We hadn’t evaluated collinearity but assessed the variance inflation factors (see Stata VIF) which are commonly used to evaluate if collinearity is an issue. VIFs above 10 signal there could be an issue, but across our factors we found that the highest VIF was 2.45 suggesting that collinearity is not an issue in our model. (See the full set below).

**Variance Inflation Factors** *(VIF>10 suggests collinearity)*

Since the factors we include are categorical, the assumptions regarding linearity are not necessary for our estimates to be unbiased. However, we checked for homoskedastic errors (ie, normality of the residuals) and found a slight skew (see the figure below) and we decided to change our model to employ Huber-White robust standard errors to account for it. While this doesn’t affect our estimates (as we noted above we believe our estimates are unbiased), it does affect the precision of our estimates and we have updated our Table 4 to reflect the “robust” model results.

**Distribution of our Table 4 Residuals before Employing Huber-White Standard Errors**

4. **In Figure 1, authors show the cumulative distribution function of the penalties across the 1000 hypothetical fiscal years for a randomly chosen hospital, which happens to be a hospital that was not actually penalized. For comparison, authors should also include a figure for a randomly chosen hospital that was penalized to see the distribution of hypothetical penalties for a penalized hospital.**

We thank the reviewer for this suggestion. We have included another figure for a randomly chosen hospital that was penalized to accompany the other in our revised manuscript.

5. **Authors state early on in the results that hospitals that are more likely to be penalized are major teaching hospitals, safety-net hospitals, those located in metropolitan areas, and those in the Northeast or South census regions, though many of these factors are associated with lower standard deviation in penalties (at least major teaching hospitals, safety-net hospitals, and the South census region), indicating less of an impact of luck. Do authors have any comment about this?**

We thank the reviewer for asking about this. We do not see any contradiction in stating these facts. These factors were associated with relatively more penalties in FY2015 (i.e., their means), and associated with more certainty (i.e., lower standard deviations). This makes a lot sense as these hospitals admit sicker, less advantaged patients (more likely to be penalized) and typically have more admission volume than other hospitals (more precise estimates in any given evaluation period).

6. **On the top of page 7, authors use "ERR" without defining it first.**

We have made this change, thanks for pointing this out.

7. **For the 1st step in the simulated penalties for 1000 hypothetical fiscal years, authors say they use regression models. I'm assuming the outcome is readmission (yes/no) in these models, so to be more clear in what was done, authors should say "logistic regression models".**

In responding to reviewer 1’s comment 3 above, we have clarified that we used linear probability models to estimate our predicted and expected readmission rates. We choose this model to deal with the computational burden of employing non-linear models and the similarity between linear and non-linear estimates when the outcome probability is between 20% and 80%, where readmission rates are just over 20% on average.

8. **On the top of page 11 "The fact we" should possibly be "The fact that we".**

We have made this change in our revised manuscript.

9. **In Table 1, authors provide the average number of beds for the non-penalized and penalized hospitals (actual and recomputed). Even though the sample size is quite large, it still would be useful to also include the standard deviation in the number of beds.**

We thank the reviewer for this suggestion. We have now included the standard deviations for average beds in our Table 1.

10. **On page 12, end of the 3rd line of the 1st paragraph, change "it" to "and" or rephrase the sentence.**

We made this change, thank you.

11. **Also on page 12, about half way down the page, authors talk about the standard deviation in penalty size for the hospital illustrated in Figure 1 as "significant variation". How do authors define "significant variation"?**

We changed this sentence to be “*sizeable…relative to the maximum penalty allowed*” to be more clear. The next sentence elaborates using an example, which we feel makes this point again and does so clearly. Please let us know, though, if you are still concerned about our revised text here.

12. **In the next paragraph (last paragraph on page 12), authors mention the average penalty size across all hospitals - is this the average penalty size when penalized?**

As we write in our manuscript, this is across all hospitals including those without a penalty. We have made this more clear in our revised draft by adding the parenthetical *“(including non-penalized hospitals)”.* While checking this section, we found that our estimates were not accurate and have updated them in the paper. This now reads:

Next, we repeat the analysis for all HRRP eligible hospitals. Across all hospitals in the analysis (including non-penalized hospitals), the average simulated penalty size was 0.78 percent of Medicare payments (1/4^th^ of the maximum penalty allowed), while the average standard deviation of penalty size was 0.89 percent of Medicare payments.

13. **On the bottom of page 15, (2nd line from the bottom), change "variation is" to "variation in"**

Thank you for catching this, we have changed it in our revised draft.

14. **In Table 4, for the 3rd column header, change "Standard deviation" to "Standard Error", since I'm assuming that is what is being presented (the standard error of the coefficient).**

Thank you for catching this, we have changed it to Standard Error in our revised draft.
